# Supplementary material for: Mutations in PCYT2 disrupt etherlipid biosynthesis and cause a complex hereditary spastic paraplegia
Source: Brain. 2019 Oct 22;142(11):3382–97. doi: 10.1093/brain/awz291 (PMC6821184; doi:10.1093/brain/awz291)
Supplement: awz291_Supplementary_Data [file awz291_supplementary_data.zip › awz291-Suppl_data/Supplementary_Data4.zip!/Supplementary information 3 - fibroblast lipidomics.zip!/Fibroblast lipidomics/Controle vs Patient2/Statistical plots and data/LPG species/Boxplots.pdf]

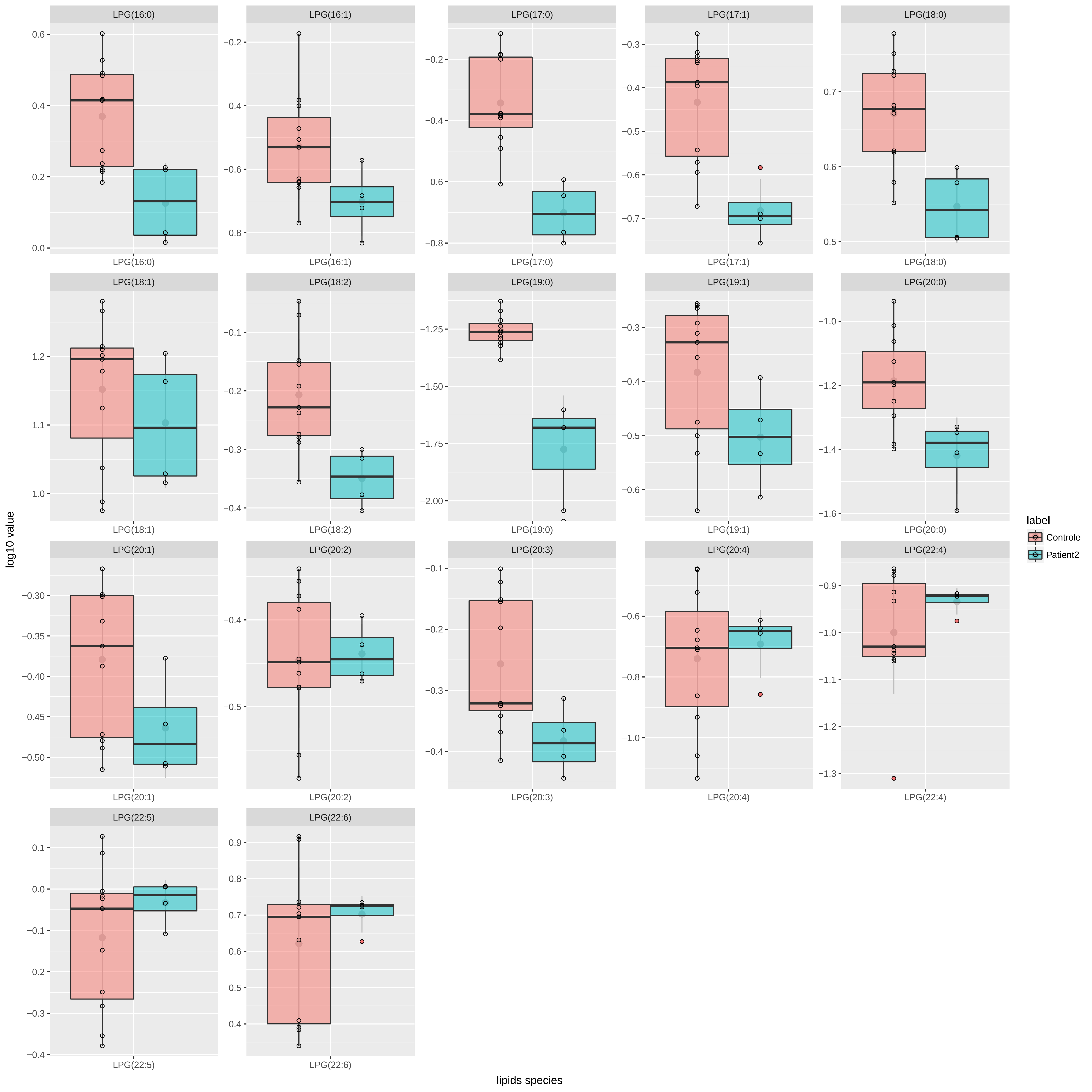

LPG(16:0)
LPG(16:1)
LPG(17:0)
LPG(17:1)
LPG(18:0)
LPG(18:1)
LPG(18:2)
LPG(19:0)
LPG(19:1)
LPG(20:0)
LPG(20:1)
LPG(20:2)
LPG(20:3)
LPG(20:4)
LPG(22:4)
LPG(22:5)
LPG(22:6)
log10 value
lipids species
label
Controle
Patient2
